# Genetic Diversity and Population Structure of Farmed Longfin Batfish (*Platax teira*) in the South China Sea

**DOI:** 10.3390/genes16111254

**Published:** 2025-10-24

**Authors:** Yayang Gao, Baosuo Liu, Huayang Guo, Kecheng Zhu, Lin Xian, Nan Zhang, Tengfei Zhu, Dianchang Zhang

**Affiliations:** 1 College of Fisheries and Life Science, Shanghai Ocean University, Shanghai 201306, China; southchinasfrigao@163.com; 2 Sanya Tropical Fisheries Research Institute, Sanya 572018, China; liubaosuo343@163.com (B.L.); guohuayang198768@163.com (H.G.); zkc537@163.com (K.Z.); 398730316@163.com (N.Z.); 3 Key Laboratory of South China Sea Fishery Resources Exploitation and Utilization, Ministry of Agriculture and Rural Affairs, South China Sea Fisheries Research Institute, Chinese Academy of Fishery Sciences, Guangzhou 510300, China; c-xianlin@genomics.cn (L.X.); zhtfsuper@outlook.com (T.Z.); 4 Guangdong Provincial Engineer Technology Research Center of Marine Biological Seed Industry, Guangzhou 510300, China

**Keywords:** *Platax teira*, genetic diversity, population structure, whole-genome resequencing, inbreeding coefficient, runs of homozygosity

## Abstract

Background: Longfin batfish (*Platax teira*) is an important economic species in southern China. In recent years, its wild population has significantly declined due to overfishing. Around 2015, breakthroughs in the artificial large-scale seedling technology for *P. teira* have promoted the growth of its aquaculture scale in regions such as Hainan and Guangdong. Methods: To study the genetic diversity, inbreeding status, and population structure of the current *P. teira* farming populations in China, we performed whole-genome resequencing technology and high-density SNP markers to analyze the genetics of four main farming populations. A total of 109 individuals from four populations (NA, ZP, XL, and XC) were sequenced, identifying 5,384,029 high-quality SNPs. Results: The results showed that the nucleotide diversity (π) of each population ranged from 0.00155 to 0.00165 and observed heterozygosity (*H*o) ranged from 0.253 to 0.282, which indicated low levels of genetic diversity. The results of the ROH analysis show significant inbreeding in the NA population. Genetic differentiation analysis revealed that the genetic differentiation among NA, XC, and ZP populations was relatively low (*F*_ST_ = 0.021–0.029). Conclusions: NA, XC, and ZP populations likely share a common origin of their fry stocks. Based on a phylogenetic tree, principal component analysis (PCA), and population structure analysis, the four populations were divided into four genetic groups. This study is the first analysis of the genetic diversity and population structure of *P. teira* farming populations in China, laying the foundation for the establishment of a base breeding population and the implementation of genetic improvement programs.

## 1. Introduction

*P. teira* (Forsskål, 1775), commonly known as the longfin batfish, belongs to the class Osteichthyes, order Perciformes, family Ephippidae, and genus *P. teira*. It is widely distributed across the Indo-Pacific region, including the Arabian Sea, Indonesia, and the Sea of Japan, and it is commonly found in the South China Sea and coastal waters of Taiwan [1]. In recent years, *P. teira* has gained increasing attention in China’s culture industry, particularly in Guangdong and Hainan Provinces, owing to its rapid growth rate, high ornamental and nutritional value, and excellent environmental adaptability. The successful establishment of *P. teira* as a new aquaculture candidate aligns with the strategic need of the Chinese aquaculture industry to diversify farmed species, enhance economic resilience, and promote sustainable mariculture.

Despite its growing importance in aquaculture, genetic studies on *P. teira* remain limited. To date, research has focused mainly on resource distribution [2], embryonic and larval development [3,4,5], muscle nutritional composition [6], and aquaculture techniques [7], while no studies have yet investigated its nuclear genome-level genetic diversity or population structure [8].

Genetic variation is fundamental to biological evolution. However, the expansion of aquaculture often involves repeated use of limited broodstock and frequent seed translocation, which may lead to inbreeding and a loss of genetic variation [9,10]. Following the onset of inbreeding, a population’s genetic variation can diminish considerably across a few generations, and restoring this lost diversity often presents a significant challenge [11]; this can negatively affect disease resistance and growth performance.

Single-nucleotide polymorphisms (SNPs) have emerged as the molecular marker of choice in population genetics studies due to their high genetic stability and lower genotyping error rates [12]. The application of single-nucleotide polymorphisms enables highly accurate and resource-effective investigations, which are fundamentally important for conducting advanced studies in genome-scale population evolution [13]. SNPs enable efficient and high-resolution analyses of genetic variation, population structure, and inbreeding across the genome. Numerous studies have successfully applied these markers to marine fish. For instance, in European sea bass (*Dicentrarchus labrax*), researchers analyzed the genetic structure of wild and cultured populations using SNP markers, revealing the genetic impact of aquaculture practices and providing valuable data for broodstock management [14]. Similarly, studies in groupers (*Epinephelus coioides*) identified genetic bottlenecks and declines in diversity in hatchery stocks, emphasizing the need for genetic monitoring [15]. Whole-genome resequencing (WGS) has emerged as a powerful tool to address these challenges, enabling high-resolution assessment of genetic diversity, inbreeding, and population structure in aquaculture species. In Nile tilapia (*Oreochromis niloticus*), WGS has revealed signatures of domestication selection and genetic erosion in farmed populations compared to their wild counterparts [16]. Similarly, in rainbow trout (*Oncorhynchus mykiss*), the use of WGS provided novel insights into the genetic diversity and structure of Sweden’s three main farmed rainbow trout populations [17]. However, a critical knowledge gap remains regarding the genome-wide genetic diversity and population structure of farmed *P. teira* populations. To date, no study has applied whole-genome resequencing to assess the genetic status of its cultured stocks.

In this research, we conducted whole-genome resequencing on 109 individual *P. teira* collected from four separate cultured populations across southern China. Our objectives were to (i) assess the genetic diversity within each population, (ii) evaluate genomic inbreeding through runs of homozygosity (ROH) analysis, and (iii) evaluate genetic differentiation and population structure among populations. Furthermore, this study serves as a valuable reference for genome-assisted breeding strategies in *P. teira* and related species.

## 2. Materials and Methods

### 2.1. Sampling and Data Collection

**Figure 1:**
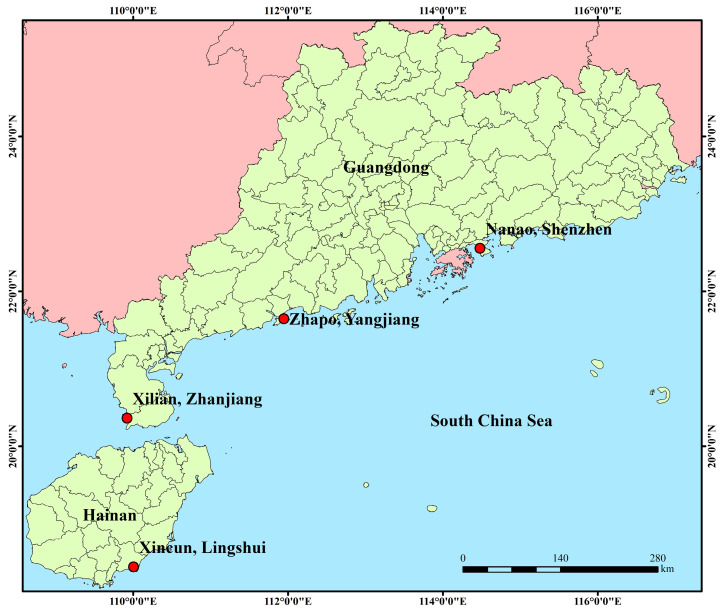
Map showing the sampling locations of the four cultured *P. teira* populations in this study. NA: Nanao, Guangdong; ZP: Zhapo, Guangdong; XL: Xilian, Guangdong; XC: Xincun, Hainan.

In this study, a total of 109 longfin batfish were collected from four distinct cultured groups on Hainan Island and Guangdong Province, China (Table 1, Figure 1). Each population was named according to its collection site, with all four originating from principal breeding farms located in the South China Sea, including NA (Nanao, Shenzhen, China), ZP (Zhapo, Yangjiang, China), XL (Xilian, Zhanjiang, China), and XC (Xincun, Lingshui, China). Fins were collected from each population in a random sampling procedure. These fins were stored in 75% alcohol at −20 °C for DNA extraction, with each group’s fins being stored separately. DNA was extracted using TIANamp Marine Animals DNA Kit (Tiangen, Beijing, China) following the manufacturer’s instructions [18]. DNA quality was determined via agarose gel electrophoresis and an Agilent 4200 Bioanalyzer (Agilent Technologies, Santa Clara, CA, USA). Sequencing of PE libraries (2 × 150 bp) was performed on the MGI-2000/MGI-T7 platform (The Beijing Genomics Institute, Qingdao, China). All experiments in this study were approved by the Animal Care and Use Committee of the South China Sea Fisheries Research Institute, Chinese Academy of Fishery Sciences (no. SCSFRI96-253), and performed according to the regulations and guidelines established by this committee.

### 2.2. Whole-Genome Resequencing and Variant Calling

Adaptor sequences and low-quality bases were filtered out from the raw reads using Fastp (v0.20.0, -n 10 -q 20 -u 40). The clean reads were mapped to the *P. teira* reference genome [19] using BWA v0.7.15 [20]. Mapping results were then converted into BAM format and sorted with SAMtools v1.3.1 [21]. The SNPs were filtered using the following criteria: (i) SNPs with QD < 2.0, FS > 60.0, MQ < 40.0, MQRankSum < −12.5, and ReadPosRankSum < −8.0; (ii) variant missing rate less than 0.1 and a per-genotype depth filter of >4 to exclude low-confidence genotype calls; (iii) minor allele frequency (MAF) > 0.01; and (iv) linkage disequilibrium pruning, performed using PLINK v1.9 [22]. Using a sliding window of 100 kb with a 10 kb step, we annotated and predicted the functional effects of the filtered SNPs based on the genome annotation file with snpEff v5.0 [23].

### 2.3. Genetic Diversity and Linkage Disequilibrium

Prior to analysis, each genotype with markers was assembled head to tail and missing sites were replaced by “-”. The parameters of genetic diversity were analyzed by VCFTOOLS v0.1.16 [24], including the expected heterozygosity (*H*e), polymorphism information content (PIC), observed heterozygosity (*H*o), nucleotide diversity (*π*), and Hardy–Weinberg equilibrium *p*-value (*HW-P*) (*p* < 0.05 indicates Hardy–Weinberg disequilibrium). We compared linkage disequilibrium (LD) patterns among different groups and LD decay was measured by calculating correlation coefficients (r^2^) for all pairs of SNPs within 500 kb using PopLDdecay v3.41 [25].

### 2.4. Genome-Wide Detection of ROH and Estimation of Inbreeding Coefficients

The PLINK v1.9 software command –homozyg was used for the detection of ROH. The parameters for ROH identification were set as follows, based on recommended practices in the literature [26]: –homozyg-kb 100 –homozyg-window-missing 5 –homozyg-window-threshold 0.05 –homozyg-window-het 3 –homozyg-window-snp 50 –homozyg-snp 50 –homozyg-density 50 –homozyg-gap 1000. The package R CMplot [27] was employed to generate visual representations of the results. The identified ROHs were classified into five distinct size categories as follows: 0.1 to 0.3 Mb, 0.3 to 0.6 Mb, 0.6 to 1 Mb, 1 to 2 Mb, and >2 Mb [28]. The total number of ROH for each animal, the length of ROH per population per chromosome, and the number of ROH per length category were calculated.

We employed the two following approaches to estimate inbreeding coefficients: F_ROH_, derived from runs of homozygosity, and F_HOM_, calculated from the comparison of observed versus expected homozygosity. F_ROH_ estimates were obtained using the R package detect RUNS v0.9.6 [29]. F_ROH_ was defined based on the proportion of the total length of the genome that is within ROH, using the total length of the *P. teira* reference genome (697.98 Mb) [30]. The individual inbreeding coefficient F_HOM_ was estimated using PLINK v1.9 with the –het option (plink –bfile dataset –het –out output_prefix).

### 2.5. Population Structure Analysis

The genetic differentiation coefficient (*F*_ST_) between geographical groups was calculated using GENEPOP v.4.5 [31]. The Reynolds’ genetic distance (DR) between geographical groups was estimated by *F*_ST_ value according to DR = −ln (1 − *F*_ST_). The neighbor-joining (NJ) tree was constructed and visualized using Mega11 software and an identity-by-state (IBS) kinship matrix, and the phylogenetic tree was further refined using the online tool iTOL (https://itol.embl.de/). Principal component analysis (PCA) based on genome-wide SNPs was conducted with PLINK, while population structure was further examined using ADMIXTURE v1.3.0 [32]. The likelihood of ancestral kinship (K) from 2 to 6 was tested using all SNPs. The IBS matrix was calculated by VCF2PCACluster V1.40 [33]. Ten different seeds were selected for 10 repeated analyses, and pong [34] was used to cluster the results 10 times according to the cross-validation error to determine the optimal K value.

## 3. Results

### 3.1. Overview of Whole-Genome Resequencing and Variant Detection

**Figure 2:**
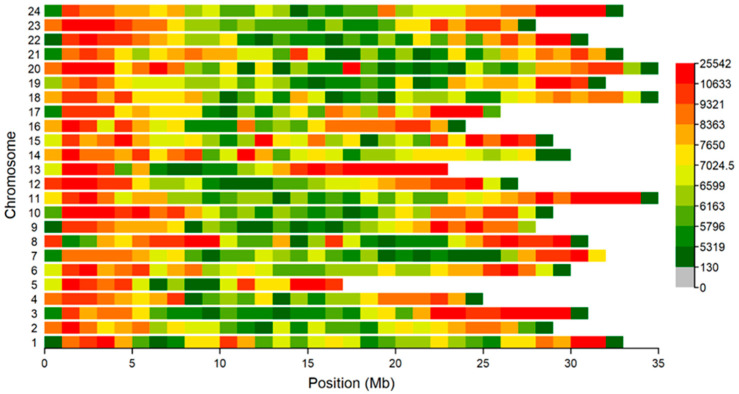
The genetic information of *P. teira*. The identification of high-quality SNPs and their distribution across 24 chromosomes of *P. teira*. The gradient colors from green to red indicate an increase in SNP density.

Whole-genome resequencing was conducted on 109 fish, generating a total of 10.68 billion reads. Of these, 99.2% successfully aligned to the reference genome using BWA, achieving an average sequencing depth of 19.5× (ranging from 13.0× to 29.3×), providing sufficient coverage for reliable downstream analyses. Following stringent quality filtering, we identified 5,384,029 SNPs across the 109 samples, with the majority residing in intronic (40.66%) and intergenic (30.74%) regions (Table 2). An examination of SNP distribution across the *P. teira* genome revealed that individual chromosomes contained between 144,120 SNPs (chromosome 5) and 275,445 SNPs (chromosome 11) (Figure 2).

### 3.2. Genetic Diversity and Linkage Disequilibrium Analysis

**Figure 3:**
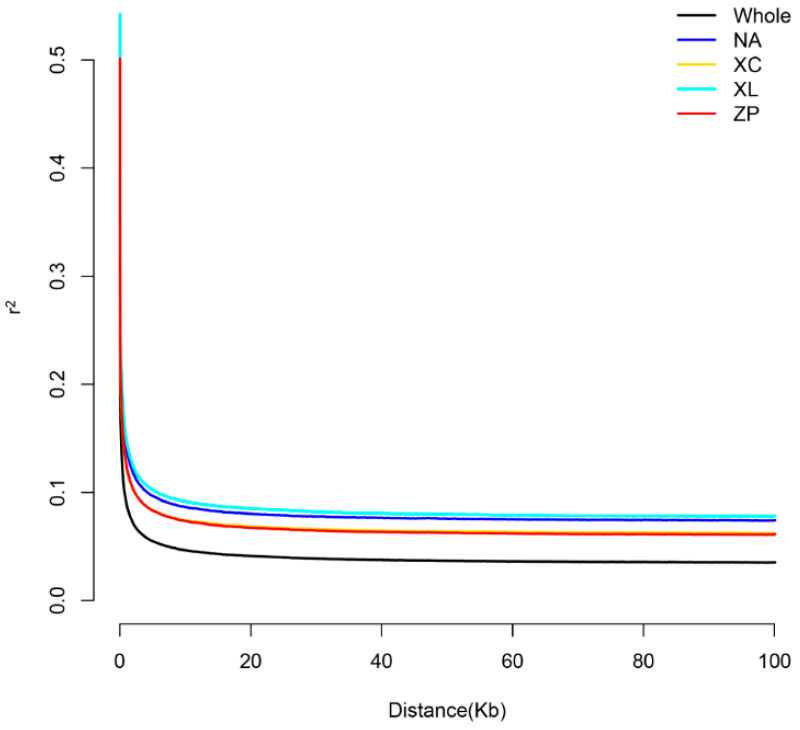
Average LD decay rates across all 24 *P. teira* chromosomes. The *y*-axis represents the average pairwise correlation coefficient (r^2^), while the *x*-axis represents the physical distance between SNPs in kilobytes (Kb). Each colored line represents the LD decay pattern of a distinct population.

We calculated the genetic diversity parameters for four *P. teira* populations based on the selected SNP loci. As shown in Table 3, Hardy–Weinberg equilibrium values (*HW-P*), observed heterozygosity (*H*o), expected heterozygosity (*H*e), polymorphism information content (PIC), and nucleotide diversity (*π*) were determined. Hardy–Weinberg equilibrium testing indicated that all farmed populations had *HW-P* values above 0.05, suggesting that genetic equilibrium was largely preserved across the *P. teira* populations. The expected heterozygosity (He) values varied from 0.242 in ZP to 0.257 in NA, and the observed heterozygosity (*H*o) ranged from 0.253 (ZP) to 0.282 (XL). (PIC) measures the level of polymorphism at each SNP site, ranging from 0.160 (XL) to 0.172 (ZP). (*π*) was consistently low across populations (0.00155–0.00165), with the highest value observed in XC (*π* = 0.00165) and the lowest in XL (*π* = 0.00155). As shown in (Figure 3), all populations exhibited some level of linkage disequilibrium, with relatively similar decay rates. The average pairwise correlation coefficient (r^2^) was higher in XL than in other reference populations.

### 3.3. Genomic Distribution of ROH and Inbreeding Coefficients

**Figure 4:**
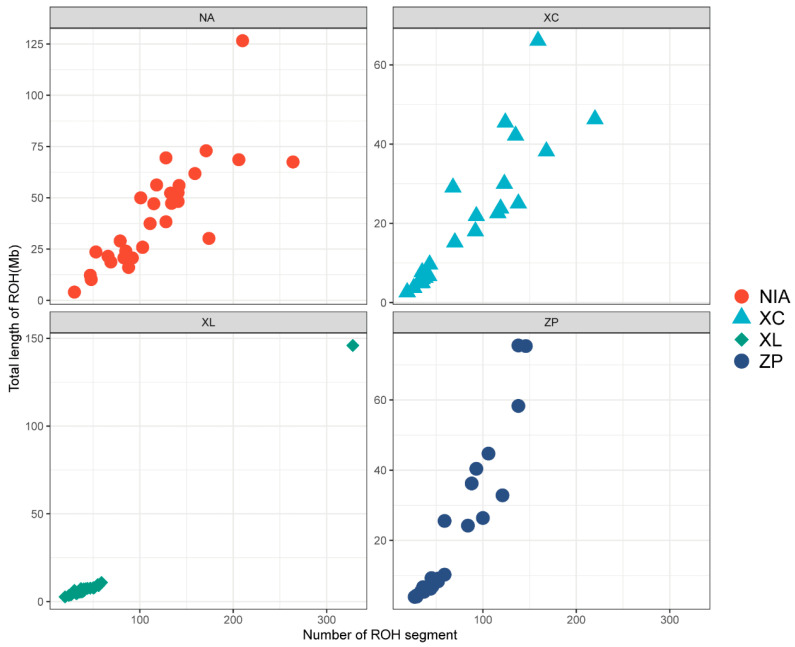
Sum number of homozygosity (ROH) of segment and total length of ROH in Mb per fish of four populations.

As shown in Table 4, NA exhibited the highest total number of ROH (3522), significantly more than ZP (1900), XC (2000), and XL (1264). All populations showed the highest proportion of short ROH (0.1–0.3 Mb; 68.3–84.3%), with XL displaying the greatest predominance of short ROH (84.3%). Notably, NA had a significantly higher proportion of long ROH segments (>0.6 Mb; 14.1%) compared to XC (6.1%) and XL (5.6%). Particularly striking was the >2 Mb ROH fraction in population NA (1.3%), while XC showed only 0.2%. These results suggest that population NA may have experienced more recent inbreeding events. The sum number of the ROH of segments and the total length of ROH in Mb per fish are shown in Figure 4. Most individuals in NA had the highest number and longest length of ROH. In contrast, the lowest number of ROH and shortest length of ROH were observed in individuals of XL.

**Figure 5:**
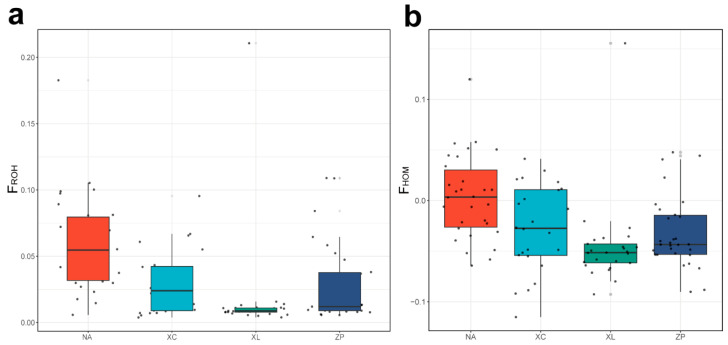
Inbreeding coefficient in four populations. (**a**) Inbreeding coefficient based on ROH. (**b**) Inbreeding coefficient based on observed and expected homozygosity.

F_ROH_ values varied from 0.0172 to 0.0594 (Table 3), with the NA population showing the highest inbreeding levels and the XL population exhibiting the lowest. Consistent with these results, F_HOM_ values followed a similar pattern across the analyzed populations (Figure 5).

### 3.4. Population Structure

**Figure 6:**
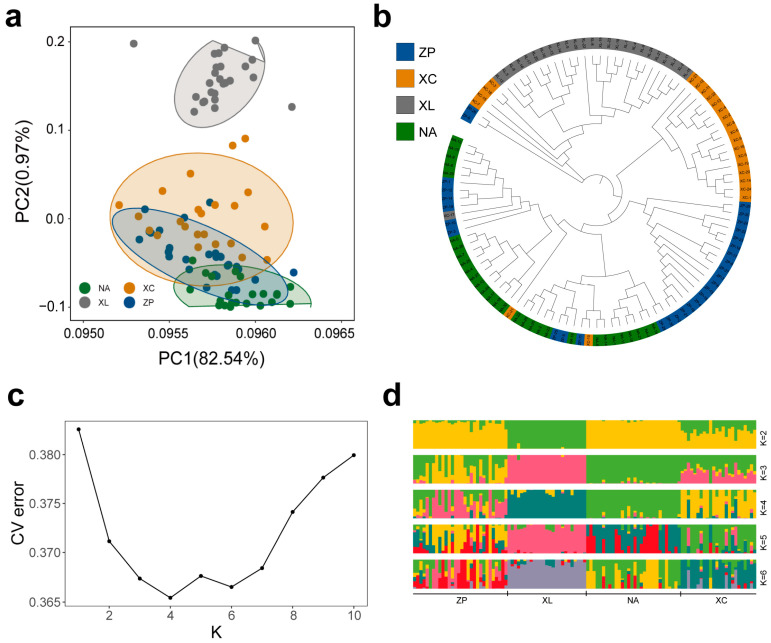
Analysis of population genetic structure. (**a**) The principal component analysis of 109 *P. teira* samples from 4 populations; (**b**) A tree was generated using the neighbor-joining method with 1000 bootstrap replicates on 109 *P. teira* samples from 4 populations. Different colors represent different populations. (**c**) Cross-validation errors in admixture analysis. X-axis represents K-values and y-axis represents corresponding cross-validation errors. Error was lowest at K = 4. (**d**) Population structure. Colors in each fragment represent the proportion of K = 2–6 ancestral populations assigned to individual genomes. Each accession is represented by a bar, and the length of each colored segment in the bar represents the proportion contributed by that ancestral population.

To evaluate the degree of genetic difference between populations, fixation index (*F*_ST_) values and genetic distances were estimated (Table 5). The highest divergence was observed between XL and NA (*F*_ST_ = 0.065) and the lowest between ZP and XC (*F*_ST_ = 0.021). PCA results (Figure 6a) show that ZP, XC, and NA were divided into three clusters with overlap. The XL distribution appears independent. In the phylogenetic tree (Figure 6b), individuals from the XC, ZP, and NA populations were extensively intermingled, forming a large cluster with poor bootstrap support at internal nodes. This pattern, indicative of high genetic similarity and recent shared ancestry, aligns with our field surveys confirming that ZP and NA initially sourced their broodstock from the XC population. Conversely, the XL population formed a distinct, well-supported monophyletic clade, confirming its independent genetic lineage. This genetic divergence was corroborated by the PCA, where XL formed a separate cluster along PC1. The Delta K results indicated that the optimal number of genetic clusters representing the most similar ancestral populations was at K = 4. The cross-validation error (CV error) reached its minimum at this value (Figure 6c), aligning with the phylogenetic tree analysis results. Moreover, the genetic structure inferred (Figure 6d) closely corresponded with the phylogenetic relationships and showed strong concordance with the clustering patterns observed in the PCA plot.

## 4. Discussion

The South China Sea represents the main aquaculture region for *P. teira*. To investigate the genetic diversity, population structure, and inbreeding status of the major cultured populations in Guangdong and Hainan Provinces, this study performed whole-genome resequencing on four populations (NA, ZP, XL, XC) from southern China. By utilizing high-density SNP markers, we provide a comprehensive assessment of the species’ genetic status.

All four populations exhibited low levels of genetic diversity, as indicated by the observed heterozygosity (*H*o: 0.253–0.282) and nucleotide diversity (*π*: 0.00155–0.00165); π values below 0.005 reflect low nucleotide diversity. Similarly, the polymorphism information content (PIC) ranged from 0.160 to 0.172 across the populations. As values below 0.25 are indicative of low polymorphism, these results collectively confirm reduced genetic variation in all four cultured populations. These results indicate that the farmed populations in this study may have been degraded to varying degrees, and effective supplementation of farmed populations are urgently needed. Low genetic diversity has also been observed in other cultured species, such as the *Coilia nasus* [35]. In all populations, *H*o was higher than *H*e (*H*o > *H*e). This phenomenon has also been observed in other farmed marine fish species, such as sea bream (*Sparus aurata*) and European seabass (*D*. *labrax*) [36]. In a finite population, it is expected that there will be random differences between the allele frequencies between both sexes, and this generates an excess of *H*o with respect to those expected with *H*e [37]. The relatively slow decay of LD in these farmed populations was most likely caused by inbreeding within each strain, although population structure may also have contributed to this. However, the potential cause of the high linkage disequilibrium observed in XL and NA might be attributed to the lower coverage depth.

The number and length of runs of homozygosity (ROH) were markedly higher in NA compared to the other populations, and the F_ROH_ values were also highest in NA (up to 0.0594), indicating strong inbreeding. ROH are reliable indicators of both historical and recent inbreeding [38]. The presence of long ROH (>2 Mb) in NA suggests recent inbreeding events, likely resulting from repeated use of close relatives or a small breeding nucleus. High levels of inbreeding and ROH accumulation have been reported in other aquaculture species, such as westslope cutthroat trout (*Oncorhynchus lewisi*) [39]; the XL population exhibited the lowest F_ROH_ and F_HOM_ values, with shorter and fewer ROH segments, suggesting lower inbreeding levels and better genetic status. This population may serve as a valuable genetic resource for future selective breeding.

An analysis of the genetic differentiation index revealed a certain degree of genetic differentiation among the populations. The genetic differentiation coefficient (*F*_ST_ = 0.065) and genetic distance (DR = 0.067) between the NA and XL populations were the highest, indicating significant genetic differences between these two populations. *F*_ST_ values between 0.05 and 0.15 represent moderate differentiation, except for NA and XL and XL and ZP populations (*F*_ST_ = 0.051); other pairwise comparisons showed moderate genetic differentiation, with *F*_ST_ values ranging from 0.021 to 0.039. Among them, genetic differentiation among XC, ZP, and NA were particularly low (*F*_ST_ values: 0.021, 0.029, and 0.026, respectively). The observed admixture patterns may reflect historical exchanges of fry among farming sites in Hainan Province; however, the exact sources remain uncertain and require further validation. According to our previous investigation, the initial breeding stock of XC originated from the wild population in the South China Sea near the Hainan Island of China. Similar trends have been reported in cultured populations of other fish (*O*. *niloticus*) [40]. In this study, genetic differentiation among three populations was particularly low, probably due to their origin from one lake. The assignment of XC, NA, and ZP in the same cluster according to both PCA and population structure analysis provides further support for the aforementioned hypothesis. In contrast, XL was relatively independent.

This study revealed the urgent need for improved genetic management in *P. teira* aquaculture. The low genetic diversity, moderate differentiation, and uneven inbreeding levels raise concerns about long-term sustainability, particularly in populations such as NA. To enhance genetic health and support future breeding programs, we recommend the following: (i) increasing broodstock size and ensure periodic introduction of wild individuals or genetically diverse stocks; (ii) establishing regional broodstock management protocols to avoid inbreeding and genetic drift; and (iii) although XL and ZP showed relatively higher genetic diversity, which makes them promising candidates for breeding programs, further validation with larger sample sizes and inclusion of wild populations is necessary before making definitive recommendations. Genomic-assisted breeding strategies, as applied in *Oreochromis*, *Salmo*, and *Sparus* species [41], can be adapted for *P. teira* to accelerate genetic improvement and maintain population resilience under intensive farming. Despite the insights provided, this study has some limitations. First, the sample size per population was limited, which may affect the accuracy of diversity and structure estimates. Second, only cultured populations were analyzed; the inclusion of wild populations would provide a more comprehensive view of domestication effects. Future research should incorporate wild populations, long-term temporal samples, and integrate phenotypic data to link genomic patterns with economically important traits.

## 5. Conclusions

This study provides a genome-wide evaluation of the genetic diversity, population structure, and inbreeding levels of four *P. teira* farmed populations from southern China. Our findings revealed generally low genetic diversity and varying degrees of inbreeding across the groups. Notably, the XL population showed relative genetic independence, while the other three populations (XC, ZP, NA) exhibited significant genetic admixture, which may be attributed to their origin—partial fry stocks were likely derived from Xincun Village in Lingshui City. The NA population showed signs of recent inbreeding, evidenced by a higher number and longer stretches of homozygous regions, while the XL population maintained relatively greater genetic variability and lower inbreeding risk. It should be noted that our sampling did not include wild populations and therefore the extent to which farming practices alone contributed to the reduced diversity cannot be conclusively determined. These results highlighted the need for improved genetic management in *P. teira* aquaculture. Strategies such as expanding broodstock sources, incorporating wild genetic material, and implementing regular genomic monitoring should be considered to maintain genetic health and breeding efficiency. By integrating genomic tools into selective breeding programs, the long-term sustainability and productivity of *P. teira* aquaculture can be better secured, contributing to both economic development and conservation of genetic resources in the South China Sea region.

## Figures and Tables

**Table 1:** Information on *P. teira* samples. Population names correspond to their collection sites, comprising NA, ZP, XL, and XC.

Location	Abbreviation	Coordinate	Sample Size
Nanao, Shenzhen	NA	114.31° E, 22.33° N	30
Zhapo, Yangjiang	ZP	111.80° E, 21.57° N	30
Xilian, Zhanjiang	XL	109.90° E, 20.42° N	25
Xincun, Lingshui	XC	109.97° E, 18.40° N	24

**Table 2:** SNP annotation by genomic region.

Genomic Region	Count	Percentage (%)
Intergenic	1,655,036	30.74
Upstream	762,891	14.17
Intro	2,189,178	40.66
Exon	207,637	3.86
Splice	19,968	0.37
Downstream	549,319	10.20
Total	5,384,029	100

**Table 3:** Genetic diversity analysis of the four populations.

Population	SNP (Num)	*H*o	*H*e	PIC	Hardy–Weinberg (HWP)	*π*	F_ROH_	F_HOM_
NA	4,225,699	0.269	0.257	0.164	0.7744	0.00158	0.059	0.003
ZP	4,667,155	0.253	0.242	0.172	0.7876	0.00164	0.028	−0.030
XL	4,224,080	0.282	0.252	0.160	0.7709	0.00155	0.017	−0.034
XC	4,625,229	0.254	0.245	0.171	0.8025	0.00165	0.029	−0.052

**Table 4:** Summary statistics of ROH in four *P. teira* populations.

	Count				Proportion (%)			
Insert Size (MB)	NA	XC	XL	ZP	NA	XC	XL	ZP
0.1–0.3 MB	2406	1615	1065	1473	68.3	80.8	84.3	77.5
0.3–0.6 MB	620	263	128	226	17.6	13.2	10.1	11.9
0.6–1 MB	254	83	40	88	7.2	4.2	3.2	4.6
1–2 MB	195	35	24	88	5.5	1.8	1.9	4.6
>2 MB	47	4	7	25	1.3	0.2	0.6	1.3
Total	3522	2000	1264	1900				

**Table 5:** Genetic differentiation *F*_ST_ value (below diagonal) and genetic distance (above diagonal) among four *P. teira* populations.

Population	NA	ZP	XL	XC
NA	-	0.026	0.067	0.029
ZP	0.026	-	0.052	0.021
XL	0.065	0.051	-	0.040
XC	0.029	0.021	0.039	-

## Data Availability

The original contributions presented in this study are included in the article. Further inquiries can be directed to the corresponding author.
